# New Insights on Factors Limiting the Carrier Transport in Very Thin Amorphous Sn-Doped In_2_O_3_ Films with High Hall Mobility

**DOI:** 10.1186/s11671-019-2948-4

**Published:** 2019-04-02

**Authors:** Yutaka Furubayashi, Makoto Maehara, Tetsuya Yamamoto

**Affiliations:** 1 Materials Design Center, Kochi University of Technology, Tosayamadacho-Miyanokuchi 185, Kami, 782-8502 Japan; 2 Industrial Equipment Division, Sumitomo Heavy Industries, Ltd., Soubiraki-cho 5-2, Niihama, 792-8588 Japan

**Keywords:** Tin-doped indium oxide, Amorphous film, Hall mobility, Mass density, Mean free path

## Abstract

We demonstrated that a mass density and size effect are dominant factors to limit the transport properties of very thin amorphous Sn-doped In_2_O_3_ (*a*-ITO) films. *a*-ITO films with various thicknesses (*t*) ranging from 5 to 50 nm were deposited on non-alkali glass substrates without intentional heating of the substrates by reactive plasma deposition with direct-current arc discharge. *a*-ITO films with *t* of more than 10 nm showed a high Hall mobility (*μ*_H_) of more than 50 cm^2^/V s. For 5-nm-thick *a*-ITO films, we found that *μ*_H_ was as high as more than 40 cm^2^/V s. X-ray reflectivity measurement results revealed that the mass density (*d*_m_) determined the carrier transport in *a*-ITO films. For *a*-ITO films with *t* of more than 10 nm, *d*_m_ had a high value of 7.2 g/cm^3^, whereas *a*-ITO films with *t* of less than 10 nm had low *d*_m_ ranging from 6.6 to 6.8 g/cm^3^. Quantitative new insight from a size effect on the carrier transport is given for *a*-ITO films with *t* of less than 10 nm. This study shows that the ratio of *t* to mean free path of carrier electrons governed *μ*_H_.

## Introduction

Sn-doped indium oxide (ITO) has been mostly applied to transparent conducting oxide (TCO) films. Indium oxide (In_2_O_3_) has a bixbyite crystal structure (space group *Ia-*3, number 206), which comprises distorted InO_6_ octahedra containing some oxygen defects. This is a periodic structure that produces structural vacancies (V_str_). Both an oxygen (O) and a structural vacancy are shared between adjacent polyhedra with the result that the polyhedra are joined at a corner occupied by the O, which is referred to as corner sharing hereafter. On the other hand, two O atoms are shared between adjacent polyhedra with the result that the polyhedral are joined along the entire edge, referred to as edge sharing hereafter. The edge-sharing structure allows a large overlap between the wavefunctions of 5*s* and 5*p* orbitals of the valence electrons of In atoms owing to the short interatomic distance of about 0.334 nm between In atoms, which should provide a high carrier mobility [1, 2]. In particular, toward widening the optically transparent range from the visible to the near-infrared spectral region for applications such as solar cells, a high Hall mobility (*μ*_H_) of more than 100 cm^2^/V s has recently been reported for hydrogenated [3] and Ce-doped hydrogenated [4] In_2_O_3_-based polycrystalline TCO films.

Most of the papers on ITO films have focused on their application as TCO films for which the typical thickness (*t*) is more than 50 nm [5]. In fact, because a TCO layer is used as an antireflection layer in a solar cell, *t* is fixed to approximately 75 nm [2]. For this value, the carrier transport properties can be described as those of a bulk material. On the other hand, there are few papers on very thin ITO films with *t* of less than 50 nm because thinner TCO films have a high electrical sheet resistance, making them unsuitable for applications. Shigesato et al. reported the electrical properties of very thin amorphous-phase ITO (*a*-ITO) films deposited by sputtering at the initial stage of growth [6]. The maximum *μ*_H_ was 40 cm^2^/V s for *a*-ITO films with *t* of 20 nm, and there was an abrupt decrease in *μ*_H_ with decreasing *t*. The initial stage of the growth of films deposited by pulsed-laser deposition (PLD) was also reported [7], where the article focused on the critical thickness and the detailed transport mechanism was not discussed.

The scattering mechanisms which include grain boundary and intragrain scattering mechanism originated by various scattering centers such as phonons, ionized impurities, and neutral impurities have been discussed for degenerated polycrystalline ITO films [8]. In contrast, for *a*-ITO films with no grain boundaries, the randomness of the In–O polyhedral-based network with short-range order should be taken into account. A preliminary analysis of amorphous zinc-doped In_2_O_3_ (*a*-IZO) films was reported [9] that was based on a defect model [10]. Utsuno et al. investigated the bonding states of both *a*- and crystalized In_2_O_3_ by a simulation analysis of grazing incidence X-ray scattering [11]. Buchholz et al. focused on the mass density of *a*-In_2_O_3_ films [12]. However, a comprehensive understanding of the dominant factors limiting carrier transport in *a*-In_2_O_3_-related systems, particularly very thin films, is still lacking because there has been no report directly showing the origin of the scattering factors.

In this work, we used ion plating with direct-current (DC) arc discharge of which product name is reactive plasma deposition (RPD) that has been commercially employed [13]. RPD with a high growth rate [14, 15] enables the fabrication of films with a uniform spatial distribution of *t* prepared on large substrates with a size such as 1.5 × 1.5 m^2^. In addition, we have recently fabricated a dense ZnO film with a thickness of 10 nm [16]. The use of RPD is thus expected to enable a reliable study of the carrier transport in very thin *a*-ITO films toward achieving high *μ*_H_ TCOs.

In this paper, we report the successful fabrication of very thin TCO films (*t* < 50 nm) based on *a*-ITO films with a high *μ*_H_ by using RPD. We found that the mass density (*d*_m_) is the most important factor for describing the carrier transport properties of the *a*-ITO system. We also reveal the relationship between *μ*_H_ and *d*_m_.

## Method

**Fig. 1:**
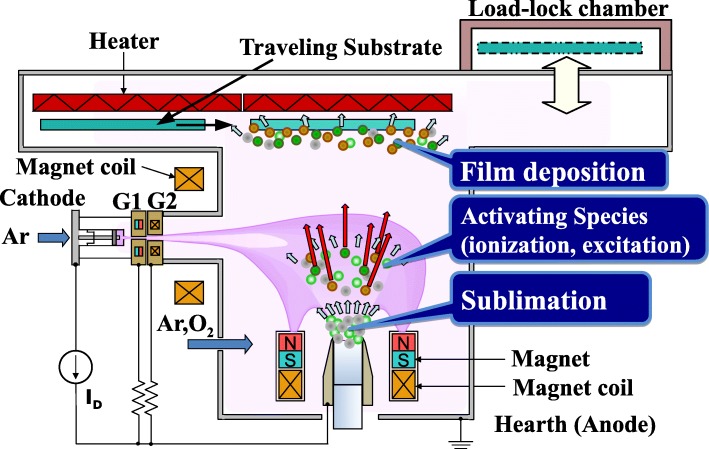
Schematic diagram of RPD with DC arc discharge

ITO films were grown on non-alkali glass substrates (Corning Eagle XG) using RPD apparatus (Sumitomo Heavy Industries, Ltd.) shown in Fig. 1. The exposure of arc plasma of electropositive argon (Ar^+^) ions and electrons generated by the pressure gradient Uramoto gun [17] to a source material made from In_2_O_3_ with a 5 wt.% corresponding to 4.6 at.% content of SnO_2_ leads to the sublimation of the source. Subsequently, some of the vaporized atoms such as In, Sn, and O change to electropositive ions such as In^+^, Sn^+^, and O^+^ ions, respectively, as results of the interactions with electrons. The source material pressed with a cylindrical form (height of 40 mm and a diameter of 30 mm) and sintered was used. The flow rates of Ar gas introduced into the deposition chamber and into a plasma gun were 25 and 40 sccm, respectively. The *a*-ITO films with *t* ranging from 5 to 50 nm were fabricated with an oxygen (O_2_) gas flow rate (OFR) of 20 or 30 sccm without intentional heating of the substrate (the substrate temperature was of less than 70 °C as a result of arc-plasma exposure). The total pressure during the growth was 0.3 Pa. The typical growth rate was 3.6 nm/s. A thickness *t* was controlled by changing the traveling speed of the substrate [18].

X-ray diffraction (XRD) and X-ray reflectivity (XRR) measurements were performed with a Rigaku ATX-G diffractometer having an X-ray source of Cu-Kα (wavelength of 0.15405 nm) to determine the structural properties of *a*-ITO films. Both XRD and XRR measurements were carried out with the same 2*θ*/*ω* configuration. The roughness and thickness of the samples were evaluated from an analysis of the XRR measurement results. An auxiliary measurement of the thicknesses was performed by using Dektak 6M stylus surface profiler (Bruker Corporation). The electrical properties at room temperature were evaluated in a van der Pauw geometry by using Nanometrics HL5500PC measurement system.

RPD equipment used in this work has been employed as a mass production usage. The spatial uniformity and the reproducibility of physical properties (including transport and thickness) of fabricated films are already ensured within ± 5% [19, 20]. Note that all the data points obtained by single measurements are sufficient in reliability.

## Results and Discussion

### Mass Density of *a*-ITO Films

No peaks were detected by XRD measurements for all the sample films, which indicates amorphous-phase films. XRR is a powerful and nondestructive technique used to study *t* and *d*_m_ for *a*-ITO films. In this work, *t* and *d*_m_ were estimated by using the XRR measurement results based on a two-layer model with an *a*-ITO film surface and a rough interface (ITO/glass) [12]. Taking into account the fact that *d*_m_ derived from the critical angle of an XRR profile corresponds to the mass density near the surface of a film, in this work, we determined *d*_m_ values from the amplitude of the oscillation for the total reflection. The results enabled us to study the relationship between *d*_m_ and the carrier mobility averaged over the whole films determined by Hall effect measurements.

**Fig. 2:**
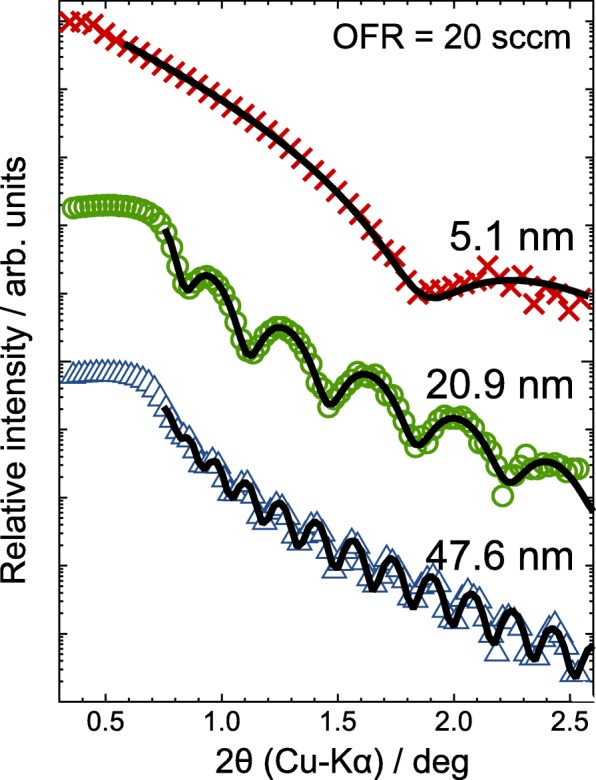
XRR data (crosses, circles, and triangles) and fitted curves (solid lines) of *a*-ITO films with thicknesses of 5.1, 20.9, and 47.6 nm grown at an OFR of 20 sccm

**Fig. 3:**
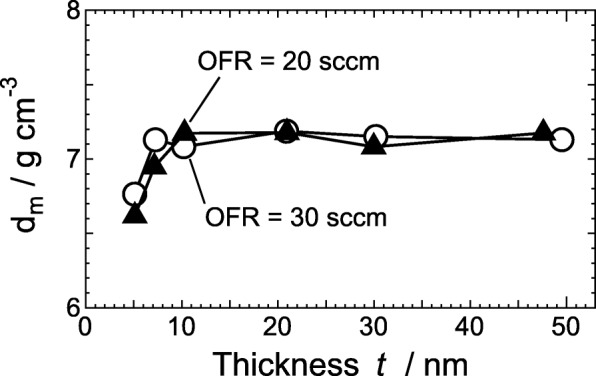
Mass density *d*_m_ derived from XRR measurement results of *a*-ITO films grown at an OFR of 20 sccm (triangles) or 30 sccm (circles) as a function of film thickness *t*

Figure 2 displays XRR spectra of *a*-ITO films with *t* of 5.1, 20.9, and 47.6 nm grown at an OFR of 20 sccm. For all the *a*-ITO films, the measured XRR curves were very well fitted by the two-layer model, as shown by the black solid curves in Fig. 2. Table 1 summarizes *t*, *d*_m_, surface roughness *r*_s_, and interface roughness *r*_i_ for *a*-ITO films determined by the XRR measurements. The thickness *t* of all the ITO films had a good agreement with those estimated by a stylus surface profiler. The values of *r*_s_ and *r*_i_ were around 1 nm irrespective to *t* and OFR. Figure 3 also shows *d*_m_ with an accuracy of ± 0.1 g/cm^3^ [21] as a function of *t*, which was evaluated from the XRR measurements. The *a*-ITO films with *t* of more than 10 nm exhibited *d*_m_ of about 7.2 g/cm^3^, which was almost the same as that of bulk ITO [12]. The *d*_m_ for *a*-ITO films with *t* below 7 nm decreased abruptly with decreasing *t* regardless of the OFR; the *d*_m_ values of 5-nm-thick *a*-ITO films with OFRs of 20 and 30 sccm were 6.6 and 6.8 g/cm^3^, respectively.

**Table 1:** Thickness (*t*), mass density (*d*_m_), and surface and interface roughness (*r*_s_ and *r*_i_, respectively) for *a*-ITO films determined by XRR measurements

Film #	OFR (sccm)	*t* (nm)	*d*_m_ (g cm^−3^)	*r*_s_ (nm)	*r*_i_ (nm)
1	20	5.12	6.62	0.89	0.92
2		7.14	6.95	0.89	1.04
3		10.3	7.17	0.88	1.04
4		20.9	7.18	0.84	1.06
5		30.0	7.08	0.74	1.06
6		47.6	7.17	0.77	0.90
7	30	5.10	6.76	0.92	0.93
8		7.26	7.13	0.74	0.84
9		10.2	7.08	0.76	0.84
10		20.9	7.19	0.81	1.00
11		30.2	7.15	1.10	1.53
12		49.5	7.13	0.69	0.84

### Transport Properties

**Fig. 4:**
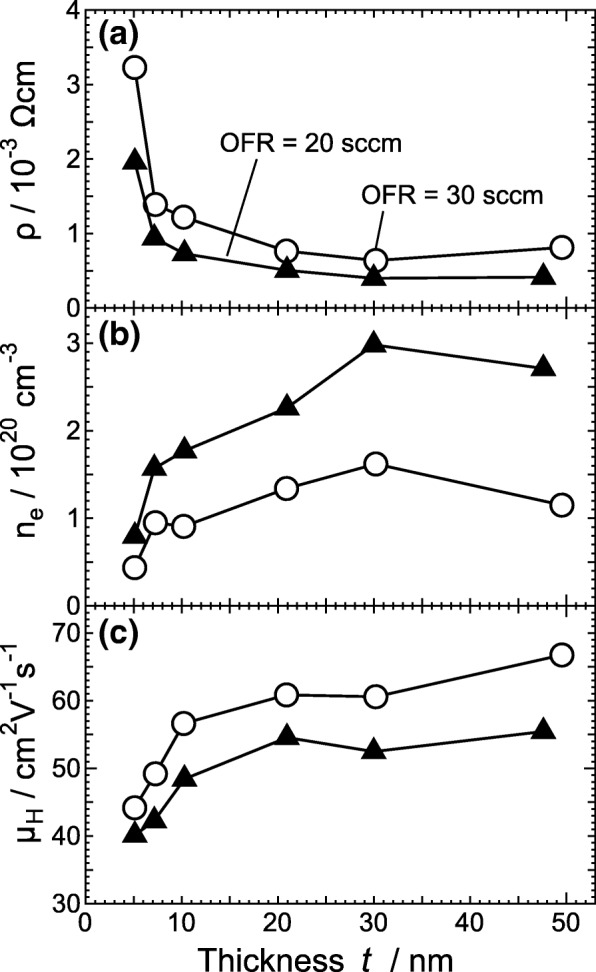
**a** Electrical resistivity *ρ*, **b** carrier concentration *n*_e_, and **c** Hall mobility *μ*_H_ of *a*-ITO films grown at an OFR of 20 sccm (triangles) or 30 sccm (circles) as functions of thickness *t*. All values were obtained at room temperature

Figure 4 shows (a) electrical resistivity *ρ*, (b) carrier density *n*_e_, and (c) *μ*_H_ for *a*-ITO films at OFRs of 20 and 30 sccm determined by Hall effect measurements at room temperature. At any given *t*, *n*_e_ for *a*-ITO films at an OFR of 20 sccm was larger than that for *a*-ITO films at an OFR of 30 sccm, whereas *μ*_H_ for *a*-ITO films at an OFR of 20 sccm was smaller than that for *a*-ITO films at an OFR of 30 sccm. This suggests that the ionized impurity scattering mechanism is one of the factors determining the *n*_e_-dependent *μ*_H_ for *a*-ITO films. The above suggested OFR dependence of *n*_e_ implies that oxygen vacancies can play a role as donor defects under the following assumptions: (1) the OFR dependence of the residual amount of Sn dopants and of the doping efficiency of the Sn donors is very small compared to the OFR dependence of the density of oxygen vacancies and (2) the density of oxygen vacancies generating shallow donor levels decreases with increasing OFR. Note that for *t* of less than 30 nm, it was found that *μ*_H_ increased with *n*_e_, which cannot be explained by conventional ionized scattering. This implies that the carrier transport is governed by another factor, such as a size effect, which will be discussed later, for *a*-ITO films.

In the case of sputtering [6] and PLD [7], the reported critical thickness was 4 nm, where a three-dimensional (3D) process turned out to be dominant and the coalescence of islands was not completed. In such films, *μ*_H_ will be significantly small around the critical thickness. For *a*-ITO films deposited by RPD, the relative decrease in *μ*_H_ at a *t* of 5 nm was less than 30% compared with that for *a*-ITO films with *t* of more than 10 nm. This suggests that RPD produces ITO films exhibiting growth via a two-dimensional (2D) process, which has already been proved for ZnO films [16].

### Dominant Features Determing *μ*_H_ for the Films: Mass Density and Mean Free Path

**Fig. 5:**
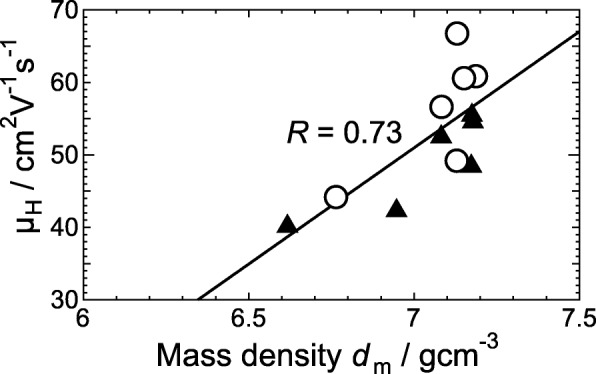
Relationship between Hall mobility *μ*_H_ and mass density *d*_m_ of *a*-ITO films grown at an OFR of 20 sccm (triangles) or 30 sccm (circles). The solid line represents a linear fit to all the data with its correlation coefficient *R* specified

**Fig. 6:**
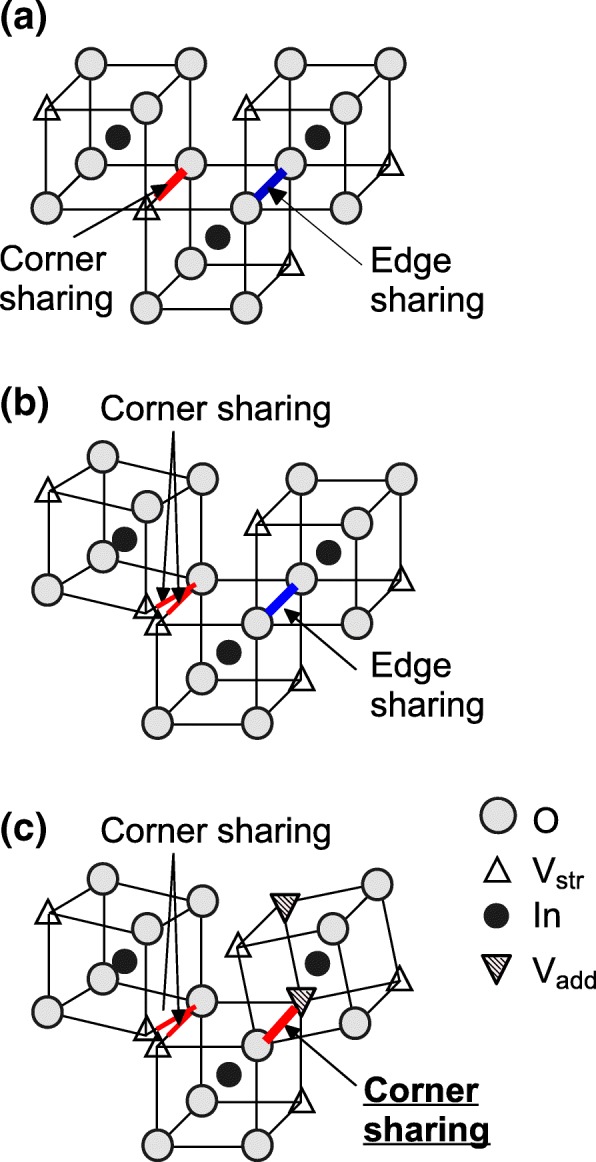
Models of local structure of **a** crystalline ITO, **b** *a*-ITO, and **c** very thin *a*-ITO with added O vacancy defects (V_add_), resulting in the transformation from edge sharing to corner sharing

Figure 5 shows the dependence of *μ*_H_ on *d*_m_ for *a*-ITO films at OFRs of 20 and 30 sccm. We found that *μ*_H_ and *d*_m_ have a strong positive correlation with its high correlation coefficient of 0.73. The results of the analysis of grazing incidence X-ray scattering by simulation suggest that *a*-In_2_O_3_ has more corner-sharing In–O–In bonds than crystalline In_2_O_3_ (Fig. 6a) [11, 12, 22]. If we assume that *a*-ITO films also have more corner-sharing In–O–In bonds than crystalline ITO films (see Fig. 6b for the model), the generation of an added vacant defect of an O atom (V_add_) in two edge-sharing O–O promotes the change in polyhedra from edge sharing to corner sharing. Subsequently, the polyhedra can rotate along an edge, thereby separating adjacent polyhedra, resulting in disjoint corner-sharing polyhedra (see Fig. 6c for the resulting model). This will result in *a*-ITO films with low *d*_m_ together with a reduced In–O coordination number, corresponding to the very thin *a*-ITO films with thicknesses of less than 10 nm. In such films, the In–In interatomic distance between the corner-shared In–O polyhedral is increased. This reduces the overlap of the wavefunctions of In valence 5*s* and 5*p* orbitals, resulting in low carrier transport together with the transformation of excess electrons provided by *n*-type defects, such as Sn substituting In atoms and O vacancies, from delocalized to localized states. We confirmed reduced *n*_e_ and *μ*_H_ for 5-nm-thick *a*-ITO films as respectively shown in Figs. 4b and c. The above discussion combined with the experimental results leads to the conclusion that the carrier transport of *a*-In_2_O_3_ films is strongly governed by *d*_m_, which determines the proportion of corner-sharing In–O polyhedra.

In addition to the above effect of *d*_m_ on carrier transport, the effects of the vertical size, i.e. *t*, on carrier mobility should be taken into account for *a*-ITO films with *t* of less than 10 nm. We estimated the mean free path of carriers (MFP; *λ*) from the transport properties shown in Fig. 4. On the basis of the Fermi gas model, the Fermi velocity of carriers, *v*_F_, can be written as *v*_F_ = (*h*/2m*)(3*n*_e_/*π*)^1/3^ [23], where *h* and m* denote the Planck constant and the effective mass of free electrons, respectively. Using the formula for carrier mobility (*μ* = *eτ*/m*, where *e* and *τ* are the elemental charge and scattering time of carriers, respectively), *λ* can be given by

$$ \lambda ={v}_{\mathrm{F}}\tau =\frac{\mu h}{2e}{\left(\frac{3{n}_{\mathrm{e}}}{\pi}\right)}^{1/3}. $$

**Fig. 7:**
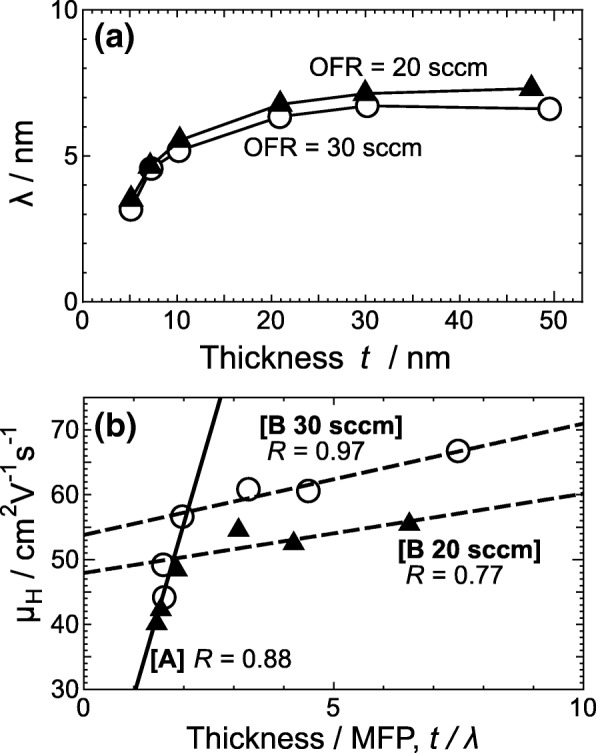
**a** Mean free path *λ* as a function of film thickness *t* and **b** relationship between Hall mobility *μ*_H_ and the ratio of thickness *t* to *λ*, *t/λ*, for *a*-ITO films grown at an OFR of 20 sccm (triangles) or of 30 sccm (circles). The solid line [A] and dot-dashed line [B; for each OFR] denote linear fits of the data for *t* = 5–10 nm and *t* = 10–50 nm, respectively. The correlation coefficients *R* are specified for all fitted lines

In this study, we took *μ*_H_ as *μ* and assumed that this model can be adopted for the *a*-ITO films. Figure 7a shows *λ* as a function of *t*. With increasing *t* up to 10 nm, *λ* increased sharply. With further increasing *t*, *λ* increased slowly, then tended to remain almost constant. This behavior of *λ* did not depend on the OFR owing to the compensation of effects between *n*_e_ and *μ*_H_. To clarify the above size effect, *μ*_H_ was plotted as a function of *t/λ*, in Fig. 7b. This relationship clearly reveals that there is a bending of a slope at *t/λ* ~ 2, which corresponds to *t* = 10 nm. The slope [A] in Fig. 7b is a fitted line for all of the data with *t* ≤ 10 nm and those two named [B 20 sccm] and [B 30 sccm] are ones for the data with *t* ≥ 10 nm, grown at OFRs of 20 and 30 sccm, respectively. It is obviously seen that those slopes have high correlation coefficients of more than 0.75. This indicates that the dependence of *λ* on the transport properties of the *a*-ITO films was found to change at *t* of 10 nm. Taking into account the fact that *λ* is comparable to *t* for very thin *a*-ITO films, we conclude that the reflection of carriers on both the surface and the interface should also be a dominant factor determining *μ*_H_.

## Conclusion

We successfully fabricated very thin *a*-ITO films with a high *μ*_H_ on glass substrates by using RPD. The relatively high *d*_m_ together with the high *μ*_H_ for a small *t* suggests almost 2D initial growth. We found that *d*_m_ is a dominant factor limiting the carrier transport of the *a*-ITO system, which is considered to be caused by the existence of corner-sharing In–O polyhedra in a matrix of edge-sharing In–O polyhedra-based network. For *a*-ITO films with *t* of less than 10 nm, the properties of the carrier transport can be characterized in terms of both *d*_m_ and *λ* for carriers. On the other hand, for *a*-ITO films with *t* of more than 10 nm, the carrier transport can be mainly described within the framework of bulk ITO without the surface or interface scattering of carriers. As the next step, we will determine the lattice structures of *a*-ITO films with various thicknesses.

## Abbreviations

2D: Two dimensional
3D: Three dimensional
*a*-In_2_O_3_: Amorphous-phase indium oxide (III)
*a*-ITO: Amorphous-phase tin-doped indium oxide
*a*-IZO: Amorphous-phase zinc-doped indium oxide
DC: Direct current
ITO: Tin-doped indium oxide
MFP: Mean free path of carriers
OFR: Oxygen flow rate during deposition
PLD: Pulsed-laser deposition
RPD: Reactive plasma deposition
TCO: Transparent conducting oxide
V_add_: Added O vacancy defect
V_str_: Structural vacancy
XRD: X-ray diffraction
XRR: X-ray reflectivity
